# Could peak proteinuria determine whether patient with dengue fever develop dengue hemorrhagic/dengue shock syndrome? - A prospective cohort study

**DOI:** 10.1186/1471-2334-11-212

**Published:** 2011-08-05

**Authors:** Farhad F Vasanwala, Rukshini Puvanendran, Stephanie Fook-Chong, Joo-Ming Ng, Sufi M Suhail, Kheng-Hock Lee

**Affiliations:** 1 Department of Family Medicine and Continuing Care, Singapore General Hospital, Bowyer Block A, Level 2, 169608, Singapore; 2 Department of Clinical Research, Singapore General Hospital, Singapore. Block 6, Level 6, 169608, Singapore; 3 Department of Renal Medicine, Singapore General Hospital, Singapore. Block 6, Level 6, 169608 Singapore

## Abstract

**Background:**

Worldwide there is a need to develop simple effective predictors that can distinguish whether a patient will progress from dengue fever (DF) to life threatening dengue hemorrhagic (DHF) or dengue shock syndrome (DSS). We explored whether proteinuria could be used as such a marker.

**Methods:**

We included patients admitted to hospital with suspected dengue fever. Starting at enrollment until discharge, each patient's daily spot urine protein creatinine ratio (UPCR) was measured. We classified those with confirmed dengue infection as DF or DHF (including DSS) based on WHO criteria. Peak and day of onset of proteinuria was compared between both groups.

**Results:**

Compared to those with DF, patients with DHF had significantly higher median peak proteinuria levels (0.56 versus 0.08 g/day; p < 0.001). For patients with DHF, the median day of onset of proteinuria was at 6 days of defervescence, with a range of -2 to +3 days after defervescence. There were three patients with DF who did not have proteinuria during their illness; the five remaining patients with DF had a median day of onset of proteinuria of was at 6 days of defervescence with a range of 0 to +28 days.

**Conclusions:**

Peak UPCR could potentially predict DHF in patients with dengue requiring close monitoring and treatment.

## Background

Dengue is the most prevalent mosquito- borne viral disease in South East Asia [1]. It is caused by four dengue virus strains from the genus Flavivirus and transmitted by the *Aedes aegypti *mosquito. The risk of severe disease and death underscores the importance of early detection of dengue fever (DF) and monitoring for signs of progression to severe disease. Currently, there are no simple clinical and laboratory markers that can predict whether a patient with DF will develop life threatening dengue hemorrhagic fever (DHF) or dengue shock syndrome (DSS) [2]. There is a need to develop a simple effective predictor that can identify patients at risk of severe disease. Ideally, the test should be cheap, fast, highly sensitive and specific.

Previous studies have documented proteinuria during the course dengue fever. In 1995, Garcia et al observed proteinuria in 22% of dengue fever patients, 38% of whom had it within the first 4 days of the onset of constitutional symptoms [3]. But the authors did not compare the occurrence of proteinuria by severity of disease. In 2004, Wills et al demonstrated among children with DSS markedly reduced plasma concentrations of different-sized proteins with a corresponding increase in fractional urinary clearances of the same proteins. They suggested that a simple test of urine protein excretion may become a useful predictor for the subsequent development of DHF and DSS [4]. In 2010, Lumpaopong et al found proteinuria in 15% of children with DF compared to 27% of those with DHF (p = 0.072) [5].

We previously reported two patients with dengue hemorrhagic fever, who developed self-limiting gross nephrotic range proteinuria without evidence of renal damage such as an increase in serum creatinine, hematuria and urinary casts [6]. The objective of this study is to assess whether proteinuria could be used to indicate which patients with dengue fever would progress to DHF or DSS.

## Methods

### Study site and population

The study was conducted at the Singapore General Hospital (SGH). SGH is the country's oldest and largest tertiary hospital and national referral center with 1521 acute-care beds. Admission criteria for suspected dengue is presence of one or more of the following: significant bleeding, severe vomiting or diarrhea that requires intravenous infusion, blood pressure < 90/60 mm Hg and/or pulse rate > 100 beats/minute, severe abdominal pain, dehydration with electrolyte abnormalities and/or postural hypotension, are elderly with medical co-morbidities and are unwell, hematocrit > 50% or platelet count < 80,000 cells/mm^3^[7]. Adult patients are admitted and managed according to WHO guidelines [8]. The following working day, the patients are transferred to the Department of Family Medicine and Continuing Care, where they were prospectively enrolled into the study. Exclusion criteria were, negative confirmatory test for dengue, failure to follow study protocol and preexisting renal disease.

### Clinical and laboratory procedures

Dengue infection was confirmed by the detection of IgM antibodies using the Dengue Fever Virus IgM capture ELISA kit (Focus Technologies™, CA, USA) [9]. In addition, we used real time one step reverse transcriptase PCR (RT-PCR) following standard procedures to confirm suspected cases of dengue fever [10]. False positive cases due to cross-reactivity using the IgM blots and RT-PCR are minimal because Japanese Encephalitis incidence is very low in Singapore, and Yellow Fever has never been detected in this region [11]

Patients' vital signs were measured every one to two hours. Postural blood pressure was taken three times daily. Patients with a postural drop in blood pressure of at least 20 mmHg in systolic and 10 mmHg in diastolic pressure readings from the lying to the standing positions were aggressively hydrated based on intravenous administration of sodium chloride 0.9% given initially at a maximum of 10-20 ml/kg/hour. If clinical improvement is observed, successful reduction of intravenous fluid 10 to 6 and 6 to 3 ml/kg/hour is made till clinical improvement is sustained i.e., hematocrit falls, pulse rate and blood pressure stable, and urine output rises. Patients with postural hypotension were examined twice daily especially for evidence of petechiae, purpura, ecchymoses, bleeding from mucosa, gastrointestinal tract or other sites.

Daily platelet counts and hematocrit were measured. Chest x-ray and/or ultrasound of the abdomen were conducted to verify pleural effusion, ascites or other signs of plasma leakage as clinically indicated. Electrolyte levels, liver function and renal function tests were carried out as clinically indicated.

### Urine protein creatinine ratio

Starting at enrollment until discharge, each patient's daily spot urine protein creatinine ratio (UPCR) was measured. As the normal physiologic amount of protein excreted is less than 0.20 gm/mg per day, we consider insignificant proteinuria as 0.06 to 0.19 gm/mg and significant proteinuria as 0.20 gm/mg or greater [12]. Patients who continued to have proteinuria at discharge were followed-up to confirm normalization of his or her UPCR.

### Definitions and data analysis

We used WHO clinical criteria to distinguish DHF from DF by the presence of hemorrhagic manifestations, thrombocytopenia and hemo-concentration [8]. Hemo-concentration is defined as an increase of the hematocrit of 20% or more; or evidence of plasma leakage such as pleural effusion, edema and ascites. Thrombocytopenia is defined as platelet count less than 100 × 10^9^/L. DHF cases with circulatory failure (evidenced by a rapid and weak pulse, narrowing of pulse pressure to < 20 mm Hg or hypotension) or profound shock with undetectable blood pressure and pulse were classified as DSS.

The onset of the illness was defined as the first day of fever. The day of defervescence (DDf) was defined as the number of days from the onset till the remission of fever. For the analysis, onset of proteinuria, the day of peak UPCR, and day of lowest platelet count, was rescaled from the DDf, considering DDf as day 0.

Statistical analysis was performed using SPSS software version 17 (IBM Corporation, Somers, NY, USA). Median, together with range, was used to describe continuous variables (UPCR, platelet count, day of peak UPCR, and day of onset of proteinuria, and hematocrit on the day of peak UPCR. The data were compared between the groups with DF and DHF (including DSS) using Fisher t-test and the Mann-Whitney's U-test, respectively. Categorical data were presented as frequency, with percentages shown in parentheses and were analyzed using Chi-square test. Statistical significance was taken as p-value < 0.05.

### Ethics

Ethical clearance was obtained from the SingHealth Centralized Institutional Review Board. (CIRB reference 2009/051/E). Waiver of consent was approved by the IRB as the collection of urine samples is non-invasive and did not constitute harm to the patient. Data were anonymized at entry and analysis. The patients were informed that the urinalysis was used to quantify the amount of protein during their illness.

## Results

**Figure 1:**
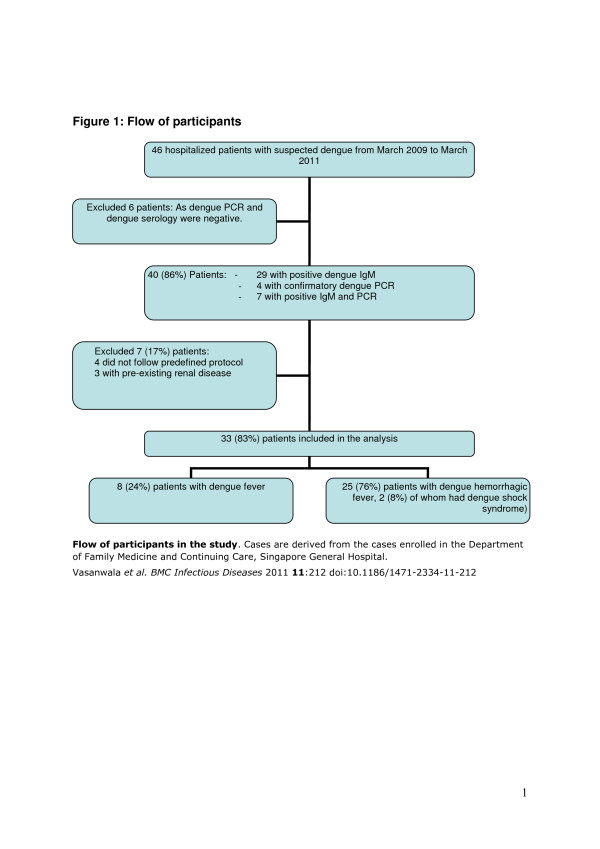
**Flow of participants in the study**. Cases are derived from the cases enrolled in the Department of Family Medicine and Continuing Care, Singapore General Hospital.

From March 2009 to March 2011, 46 patients admitted with suspected dengue infection were admitted (Figure 1). Six patients were negative for dengue serology and PCR, four patients did not follow the study protocol as described and three patients had preexisting renal disease. These 13 (17%) patients were excluded from the study. Among the 33 (83%) patients studied, eight cases (24%) were classified as uncomplicated dengue fever (DF) and 25 (76%) cases as DHF, including two cases with DSS (Figure 1).

Demographics of patients with DF and DHF are shown in Table 1. Patients with DF were significantly younger compared to those with DHF (34 ± 12 vs. 43 ± 11 years, p = 0.05). There was no difference in the level of hematocrit between these two group of patients as these patients were aggressively hydrated during admission, hence the hematocrit levels falls rather than rises during admission [7].

**Table 1:** Characteristics of patients with dengue fever (DF) and dengue hemorrhagic fever (DHF), including dengue shock syndrome (DSS)

	Dengue (N = 8)	DHF/DSS (N = 25)	P value
**Mean (± SD) range in years**	34 (12)	43 (11)	0.058

**Median (range) number of days of illness prior to admission**	6(3,9)	5(2,7)	0.190

**Median (range) DDF^1 ^in days**	6(0,10)	6(3,7)	0.789

**Median (range) number of total days of illness**	9(6,10)	9(7,12)	0.220

**Median (range) platelet count in 10^9^/L at the day of peak proteinuria**	70 (43,118)	41(14,149)	0.019

**Median (range) of hematocrit in % during the peak of proteinuria**	40.6 (38,48)	41.2 (32,48)	0.914

^1^DDF: day of defervescence from the onset of the illness

^2^The hematocrit is not significantly different due to aggressive intravenous hydration when the patients were admitted. Hydration affects the hematocrit values. The degree of intravenous hydration depends whether the patient has DF or DHF/DSS.

Table 2 shows the proteinuria and platelet parameters of patients with DF and DHF. Compared to those with DF, patients with DHF had significantly higher median peak UPCR levels (0.56 gm/mg vs. 0.08 gm/mg; p < 0.001). 96% (24 of 25) of patients with DHF developed significant UPCR, compared to none with DF developing significant proteinuria (3 out of 8 had non detected UPCR below 0.06 g/dl) (χ^2 ^= 28.2, p = 0.0005). For patients with DHF, the day of proteinuria-onset in DHF group was closer than that of DF group (I, -2 to +3 day vs. 1, 0 to +9 day in DF, p = 0.02). 19 (76%) of 25 patients with proteinuria that were followed up, had normalization of UPCR. The rest was lost to follow up.

**Table 2:** Proteinuria and platelet parameters of patients with dengue fever (DF) and dengue hemorrhagic fever (DHF), including dengue shock syndrome (DSS)

	DF (N = 8)	DHF/DSS (N = 25)	P value
**Median (range) peak proteinuria in gm/mg**	0.08 (0.06,0.15)	0.56 (0.13,6.08)	< 0.001

**Median (range) day of onset of proteinuria^1^**	6 (0, +28)	1 (-2, +3)	0.020

**Median (range) day of peak proteinuria^2^**	6 (0, +28)	1 (0, +3)	0.107

**Median (range) nadir platelet count^2 ^in 10^9^/L**	74 (43,97)	33(11,103)	< 0.001

**Median (range) day of nadir platelet count^3^**	0 (-2,+2)	1(-1,+3)	0.204

**Median (range) platelet count in 109/L at the** **day of peak proteinuria**	70 (43,118)	41(14,149)	0.019

**Number of significant UPCR^4^**	0	24(96%)	

**Number of non significant UPCR^4^**	8(100%)	1(4%)	

^1^Day of onset of proteinuria: Occurrence of proteinuria on the time scale "...-3, -2, -1, 0, +1, +2, +3," wherein "0" corresponds to the day of defervescence.

^2^Day of peak proteinuria: Occurrence of maximum proteinuria on the time scale "...-3, -2, -1, 0, +1, +2 +3," wherein "0" corresponds to the day of defervescence.

^3^Day of nadir platelet count: Occurrence of lowest platelet level on the time scale "...-3, -2, -1, 0, +1, +2, +3,...." wherein "0" corresponds to the day of defervescence.

^4 ^X^2 ^= 28.2, p < 0.005.

## Discussion

In our series, patients with uncomplicated DF did not develop significant proteinuria, while 96% of patients with impending dengue hemorrhagic fever and dengue shock syndrome developed that. In addition, they developed maximum proteinuria near the day of defervescence. This corresponded to the lowest platelet count as well that developed around the same period as the constitutional symptom i.e. fever disappeared [13]. We found a statistically significant association of higher degree of thrombocytopenia with significant proteinuria (Table 2). The degree of thrombocytopenia also corresponds to the severity of other types of hemorrhagic viral fever [14]. However we noted that proteinuria is self limiting and resolves on resolution of the illness.

Epidemiologically dengue fever is a frequently occurring illness in children and adult of the developing nations in Asia, Africa and South America with an increasing morbidity and mortality because of hemorrhagic complication or dengue shock syndrome [15-17]. Complicated dengue carries a higher mortality and morbidity rate in both pediatric and adult population. Early prediction and detection of dengue complication could enable physicians manage the complicated dengue cases with a better strategy for preventing mortality and morbidity. In a recent retrospective study, Lima et al, showed that detection of NS1 antigen of dengue virus (DENV) in tissues of dengue fatal cases had a strong positive association with confirmation of previously fatal cases of dengue fever. Even though, this DENV NS1 capture assay was studied as a test for a rapid and valuable postmortem dengue diagnostic test, they postulated that, with an increasing number of DHF and associated fatality, the availability of this new approach would be useful for the disease surveillance [18]. Although this is an approach for early diagnosis of DHF by antigen assay, it may not be practicable in clinical practice as it is a tissue diagnosis based on postmortem tissue analysis of fatal cases.

Association of proteinuria in dengue has been known to investigators for sometime. But, they could not find any difference in proteinuria between DF and DHF [5]. Unlike our study, these studies did not differentiate between significant and non-significant proteinuria. We could not identify in literature any study measuring daily UPCR for detecting peak proteinuria, as one sample of spot UPCR may not be representative of peak proteinuria for the representative case. These could explain the observation of lack of difference in proteinuria between DF and DHF in those studies.

The importance of proteinuria resulting from dengue induced secondary glomerulonephritis is elucidated in literature by various authors [19]. Renal biopsy in DHF, even showed the presence of IgA nephropathy that was transient in nature, showing the possibility of immune-complex disease in dengue fever because of DENV associated antigen-antibody-complex deposition in renal glomerular tissue [20]. Identification of soluble immune complex in DHF specific for dengue virus in serum reiterates this issue [21]. Antibodies to DENV were known to be associated with DHF in earlier studies as back as 1969 [22]. In -vitro studies with normal human B-lymphocytes demonstrated the detection of immune complex with preformed dengue antigen. This indicates that dengue immune complex might deposit in vascular and glomerular tissue leading to vasculitis of DHF and glomerulnephritis leading to proteinuria [23]. The degree of proteinuria (peak UPCR as studied in our series) might indicate the severity of dengue infection. The significant peak proteinuria could be a manifestation of an autoimmune pathogenetic mechanism that the virus triggers on the lympho-reticular system, resulting in glomerular leakage of protein due to glomerulonephritis associated with DHF [6]. Thus, peak UPCR could have an impact on predicting evolution of DHF. However, the glomerulonephritis itself resolves when the patient recovers from the illness.

Limitation of our study was the absence of renal biopsy to document and confirm glomerulonephritis in cases of significant UPCR as it was felt not necessary because of the self limiting nature of proteinuria. The other limitation was the lag period between admission of the case and initiation of collection of spot urine for UPCR, as urine was collected only after including the case in the study protocol after meeting the inclusion criteria. The study protocol would be initiated after the case had been transferred to study team from the admitting team. Since we measured daily UPCR for detecting the peak, this limitation might not have an impact on the final outcome. The onset of proteinuria could have been earlier in fact. In spite of these limitations we strongly feel that the peak UPCR in dengue should be practiced by primary care physicians when they encounter suspected cases of dengue as the onset of proteinuria and subsequent demonstration of peak UPCR could have a major implication in identification of incipient DHF and stratification of management strategy for such cases. The primary physician in the community has an advantage of seeing the patients earlier. They can administer the relevant tests including the UPCR more quickly. This may better assist the physician to stratify the severity of the illness earlier on. A similar analogy would be the diagnosis of preeclampsia/eclampsia, where the onset of new onset proteinuria from a patient without preexisting renal disease is highly significant of impending disease [24].

Further studies to define a commercially available urine dipstick equitable to the UPCR for detecting urinary protein in a patient with dengue with good sensitivity and specificity would reduce the difficulty of UPCR estimation. This test with a threshold value of 0.20 gm/mg could be used as an early warning system for predicting dengue complications. Further studies with large series of patient at multi-center set-up and improved protocol of collection of urine at primary care levels, are required to validate the important positive finding of our small series. Protocol could include use of use of urine dip-stick and UPCR together to validate more convenient urine dip-stick over the complicated and laboratory dependent UPCR.

## Conclusion

We conclude that the onset and the peak of proteinuria using the UPCR have a significant association with subsequent development of DHF. Therefore, the daily UPCR could be a useful simple adjunct for physicians in the community hospital for early determination of dengue complications.

## Competing interests

The authors declare that they have no competing interests.

## Authors' contributions

FFV designed, managed, enrolled, coordinated, analyzed and drafted the manuscript. RP helped in the design, enrolment, analysis, and drafting of the manuscript. SFC helped in the design and analysis of the data. MNJM helped in the enrolment, and drafting of the manuscripts. SMS helped in the analysis and editing of the manuscript. LKH helped in the enrolment, and analysis of the manuscript.

All authors have read and approved the final manuscript.

## Pre-publication history

The pre-publication history for this paper can be accessed here:

http://www.biomedcentral.com/1471-2334/11/212/prepub
